# Visuomotor Transformation in the Fly Gaze Stabilization System

**DOI:** 10.1371/journal.pbio.0060173

**Published:** 2008-07-22

**Authors:** Stephen J Huston, Holger G Krapp

**Affiliations:** 1 Department of Zoology, University of Cambridge, Cambridge, United Kingdom; 2 Department of Bioengineering, Imperial College London, London, United Kingdom; 3 Division of Biology, California Institute of Technology, Pasadena, California, United States of America; Bielefeld University, Germany

## Abstract

For sensory signals to control an animal's behavior, they must first be transformed into a format appropriate for use by its motor systems. This fundamental problem is faced by all animals, including humans. Beyond simple reflexes, little is known about how such sensorimotor transformations take place. Here we describe how the outputs of a well-characterized population of fly visual interneurons, lobula plate tangential cells (LPTCs), are used by the animal's gaze-stabilizing neck motor system. The LPTCs respond to visual input arising from both self-rotations and translations of the fly. The neck motor system however is involved in gaze stabilization and thus mainly controls compensatory head rotations. We investigated how the neck motor system is able to selectively extract rotation information from the mixed responses of the LPTCs. We recorded extracellularly from fly neck motor neurons (NMNs) and mapped the directional preferences across their extended visual receptive fields. Our results suggest that—like the tangential cells—NMNs are tuned to panoramic retinal image shifts, or optic flow fields, which occur when the fly rotates about particular body axes. In many cases, tangential cells and motor neurons appear to be tuned to similar axes of rotation, resulting in a correlation between the coordinate systems the two neural populations employ. However, in contrast to the primarily monocular receptive fields of the tangential cells, most NMNs are sensitive to visual motion presented to either eye. This results in the NMNs being more selective for rotation than the LPTCs. Thus, the neck motor system increases its rotation selectivity by a comparatively simple mechanism: the integration of binocular visual motion information.

## Author Summary

Many behavioral tasks rely on sensory information. This information, however, needs to be transformed into a format that is compatible with the requirements of motor systems. In this study we characterize the neural basis of such a sensorimotor transformation in a model system. Flies, like humans, stabilize their gaze to keep their eyes level, even when the body is rotating. An identified population of fly brain neurons called lobula plate tangential cells (LPTCs) contributes to this task. These cells analyze the wide-field retinal image shifts generated when the fly is moving relative to its environment. We have characterized the visual receptive fields of motor neurons that use the information encoded by LPTCs to control gaze-stabilizing head movements. Our results suggest that the motor neurons use their LPTC inputs in a comparatively simple and direct way: they combine inputs from both sides of the brain to increase the motor neurons' selectivity for rotations. Such a mechanism enables a specific, fast, and surprisingly simple sensorimotor transformation in which visual information contributes to gaze stabilization.

## Abstract 3

Visual receptive fields of fly neck motor neurons suggest a direct and efficient way that sensory signals are transformed into motor commands.

## Introduction

The nervous system encodes high-level sensory features such as the location of sound sources [1], wind direction [2], or visually analyzed parameters of self-motion [3,4] across populations of interneurons [5]. Before the responses of these interneuron populations can be used to guide behavior, they must first be transformed into a form that is appropriate for the motor system. How is such a sensorimotor transformation achieved by the nervous system? We address this fundamental question, relevant to animals across many phyla, in an experimentally tractable model system: visual gaze stabilization in the fly.

### Fly Gaze Stabilization

The fly provides us with the opportunity to apply an integrated systems approach. Quantitative analysis of distinct behaviors may be combined with electrophysiology, neuroanatomy, neurogenetics, and computational modeling to study the neural basis of behavior [6–9]. Minimal dissection is required to access the fly visual system, allowing electrophysiology to be conducted with the neural circuitry intact. Lesion and neurogenetic experiments conclusively show that a population of individually identified interneurons in the fly's third visual neuropil, the lobula plate, are key to optomotor behavior and visual gaze stabilization [10–13]. These lobula plate tangential cells (LPTCs) selectively integrate local motion signals provided by retinotopically arranged arrays of directional-selective small field elements. Two LPTC subgroups, ten VS cells (vertical system [14]) and three HS cells (horizontal system [15]), in either side of the brain are major output elements of the lobula plate [16].

**Figure 1:**
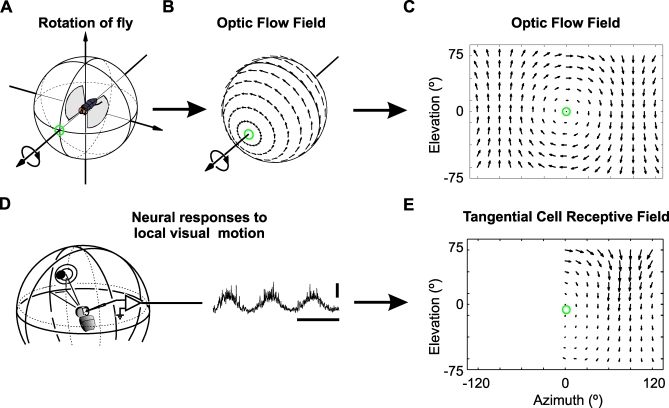
Receptive Field Maps of LPTCs Are Similar to Optic Flow The optic flow field resulting from roll-rotation to the left (A) is plotted on the visual unit-sphere surrounding the fly (B) and in a 2D cylindrical projection (C). The orientation and length of each arrow indicates the direction and magnitude of local retinal image shifts at different positions within the spherical visual field. (D) A small dot traveling on a circular path is used to characterize a LPTC's response to local motion at different positions in the fly's visual field. The response of the directional selective neuron is modulated by the changes of the dot's directional motion (for further details see [40] and Materials and Methods section). horizontal scale bar: 500 ms, vertical scale bar: 10 mV. (E) Part of the receptive field map of the tangential cell VS6 as obtained by Krapp et al. [17] using the experimental procedure shown in (D). The orientation and length of the arrows indicate the local preferred direction and relative motion sensitivity at the respective position within the cell's receptive field. The green circle marks the approximate position of the cell's preferred axis of rotation. Note that the cell's local preferred directions are oriented along concentric circles centered on the preferred axis in a manner reminiscent of the motion vectors in a roll flow field (C) and (E).

The receptive field maps of each of the VS and HS LPTCs show marked similarities to the panoramic retinal image shifts or “optic flow fields” generated during rotations of the fly about different body axes (Figure 1) [17,18]. This has been taken to suggest that the LPTCs encode information about the fly's self-rotation [17,18]. More-recent experiments have shown that LPTC responses to naturalistic stimuli contain information about both translation and rotation of the fly [19,20]. For gaze stabilization only rotation information is important, whereas translation information may be used for tasks such as distance estimation.

LPTCs convey visual information to neck motor neurons (NMNs) using direct connections and also indirectly via descending neurons [21]. NMNs combine the LPTC inputs with those from other sense organs to control head rotations that keep the retinal image level when the body rotates [22]. Because NMNs are concerned with correcting for body rotations, not translations, they must separate out the rotation-induced components of the LPTC responses from those induced by translational self-motion. The neural mechanisms underlying this task are currently not very well understood.

In a pioneering study, Milde et al. [23] showed that, like the LPTCs, some NMNs respond to visual motion in a directional-selective manner. In addition, whole-nerve stimulations suggest that the directional preferences of NMNs within a given nerve are appropriate for controlling the muscles they innervate [24,25]. Previous studies, however, did not address the question of how the self-motion parameters encoded across the population of LPTCs are used by the neck motor system. We mapped the receptive fields of NMNs in the blowfly (Calliphora vicina) to characterize the transformation occurring between the LPTC and NMN populations.

## Results

According to Strausfeld et al. [25], the fly has four known pairs of neck nerves containing a total of 21 pairs of NMNs, only a subset of which respond to visual motion with changes in spike rate. The four nerves are referred to as the frontal nerve (FN), cervical nerve (CN), anterior dorsal nerve (ADN), and ventral cervical nerve (VCN). The NMNs these nerves contain innervate 21 pairs of neck muscles. In most cases each muscle receives input from only one NMN [24,25].

**Figure 2:**
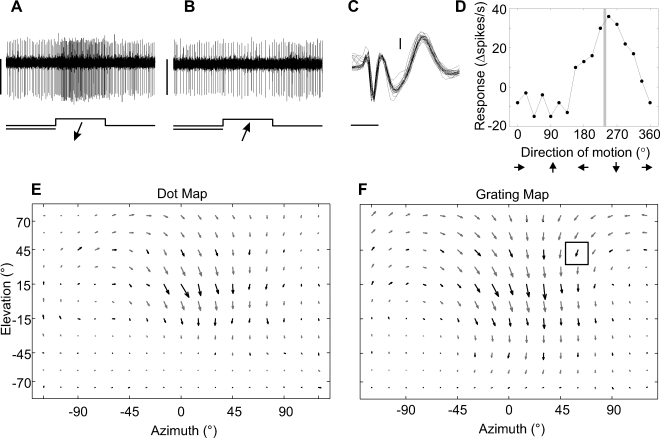
Characterizing the Visual Receptive Field of a NMN in the Left CN The responses of the NMN are shown to oblique downward (A) and upward (B) motion of a grating moving perpendicular to its orientation at 60° azimuth and 45° elevation. The bottom traces and arrows give the time courses and directions of the visual stimuli, respectively. In (A) and (B), horizontal scale bar: 1 s, vertical scale bar: 0.05 mV. (C) Extracellular hook electrode recording from a neck muscle close to the neck motor neuron, different from those shown in (A) and (B). Threshold-triggered waveforms are overlaid to show the 1:1 correspondence between a low-latency sharp waveform, most likely the neck motor neuron action potential, and the subsequent longer-latency wide waveform, most likely the muscle potential of the innervated muscle. Horizontal scale bar: 1 ms, vertical scale bar: 0.2 mV. (D) Directional tuning curve of the same cell for which individual recording traces are shown in (A) and (B). The vertical gray line indicates the local preferred direction defined by the phase of the first harmonic obtained from the tuning curve's Fourier transform. (E) and (F) Maps of the local preferred directions and motion sensitivities obtained at many different positions with the “dot method” (Figure 1D) and the “grating method”. The boxed arrow in (F) is derived from the directional tuning curve in (D). Black arrows indicate experimental results; grey arrows were obtained by interpolation (see Materials and Methods section for further explanation).

Using extracellular recordings, we determined the local directional preferences (Figure 2) within the visual receptive fields of 47 wide-field NMNs in 38 flies. We defined wide-field NMNs as those that responded to our visual stimulus over a range of positions that was equal to or greater than 90° along the azimuth. The recordings were primarily taken from the nerves themselves and in some cases from the muscles that the NMNs innervate. When recording from the muscles, it was possible to isolate a single NMN's extracellular action potential waveform, but it was not possible to identify the nerve through which the unit was running with 100% confidence, as it is in nerve recordings. In these situations, we determined the nerve identity of the unit from the position of the electrode relative to the muscles and the known NMN-muscle connectivity [25]. In such cases where the nerve that a unit belongs to was determined from the muscle recording site, the nerve to which it was attributed is marked with an asterisk

Most neck muscles are thought to receive only one NMN input [25]. In those recordings where we placed the electrodes near a NMN's innervated muscle, a slow waveform, most likely the muscle potential, could be seen to follow the NMN action potential in a 1:1 fashion (Figure 2C). Thus, many of the NMN receptive field maps may also indicate the visual receptive field of the muscles the NMNs innervate. To our knowledge, this is the first time the visual receptive fields of muscles have been obtained.

### Visual Receptive Fields of NMNs

**Figure 3:**
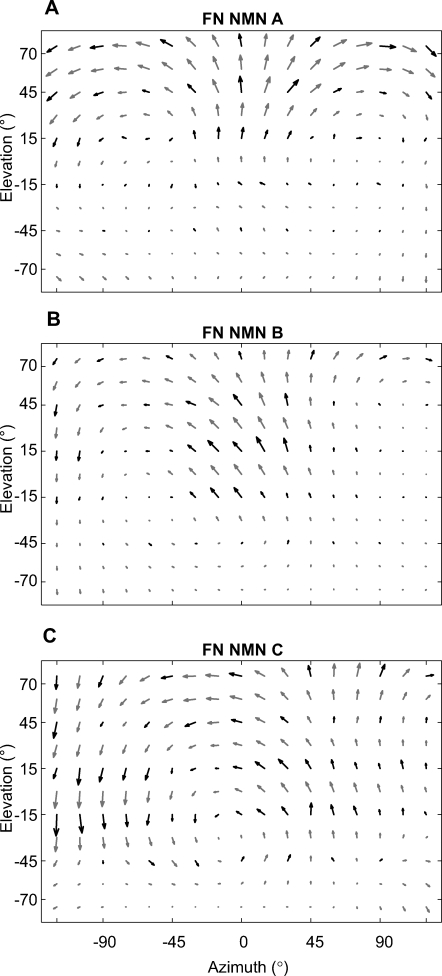
Receptive Field Maps for NMNs of the FN Black arrows indicate experimental results; gray arrows were obtained by interpolation. All receptive fields are transformed to appear as if they were taken from the left FN and were obtained using the grating stimulus. The change of local preferred directions within these maps is reminiscent of specific optic flow fields generated during pitch (A), a combination of pitch and roll (B), and almost pure roll rotation (C).

**Figure 4:**
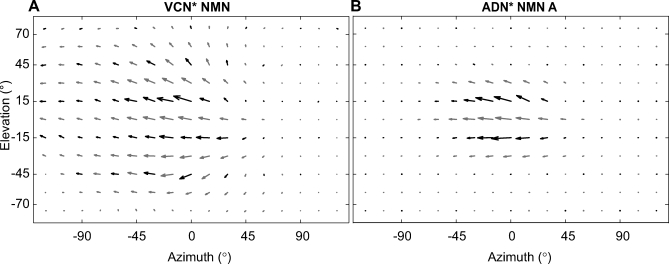
The Receptive Field Maps of Two NMNs That Mainly Respond to Horizontal Motion Both receptive fields are transformed to appear as if they were recorded from the left side of the nervous system. (A) The receptive field of a VCN^*^ NMN recorded from the OH muscle group. (B) A small ipsilateral receptive field of an ADN^*^ NMN that extends to nearly 90° along the azimuth. This NMN was recorded from the TH muscle group. In both (A) and (B) the asterisks indicate that the nerve assignment is based upon the NMN-muscle connectivity [25] and not on a direct recording from the nerve itself (see results). All data were obtained using the grating stimulus.

**Figure 5:**
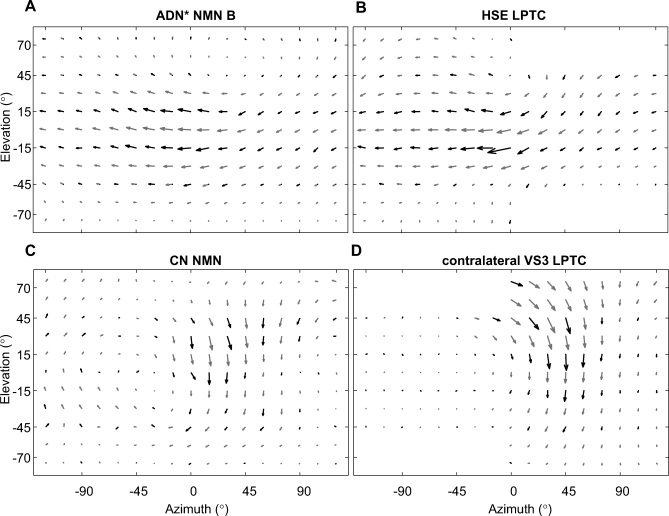
Comparison of NMN and LPTC Receptive Field Maps (A) The receptive field of an ADN* NMN, obtained using the grating stimulus. The recording was made from the TH muscle group. The known NMN-muscle connectivity [25] strongly suggests that this unit belongs to the ADN. The asterisk indicates that this nerve assignment is based on anatomical criteria. The receptive field of this NMN is similar to that of an identified HSE tangential cell shown in (B). Both the ADN NMN and the HSE LPTC have receptive fields reminiscent of the optic flow field induced by yaw rotation. HSE data were taken from [29]. (C) The receptive field obtained from a NMN in the left CN, using the dot stimulus, is similar to that of the contralateral identified VS3 tangential cell shown in (D). Both the CN and VS3 receptive fields are similar to an optic flow field generated during nose-up banked turn to the left (for further explanation see text). VS3 data were taken from [27].

**Figure 6:**
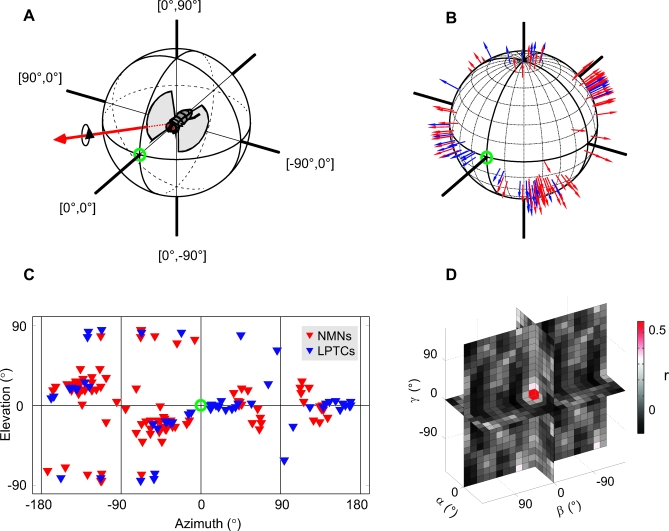
Comparison of the Distributions of Preferred Axes of Rotation for NMN and LPTC Populations (A) A fly aligned with the coordinate system we use to plot the data. The fly faces the green circle at 0° azimuth, 0° elevation. An example preferred axis of rotation is plotted as a red arrow to illustrate our plotting convention. The fly would have to rotate clockwise about the axis to maximally stimulate the cell whose preferred axis is represented by the red arrow. (B) The preferred axes for all NMNs (red) and LPTCs (blue) plotted using the same convention as in (A). To account for the contralateral counterparts of the NMNs and LPTCs we duplicated all the receptive fields, mirror-transformed them about the *y*-axis and re-estimated the preferred axes for the transformed receptive fields. Note that because we only plot the clockwise portion of the preferred rotation axis, the duplication does not result in symmetry across the azimuth = 0° line in our plot. (C) 2D cylindrical projection of the same data presented in (B); note that the 2D projection allows visualization of all the data, but introduces distortions so that the tightly packed clusters at the top and bottom appear spread out. See Figure S1 for information on which preferred axis corresponds to which cell type. (D) Normalized spherical cross-correlation [30] between the two axis distributions shown in (B) and (C). The color of each square gives the correlation coefficient between the NMN and LPTC axis distributions shown in (B) and (C), given a relative rotation between the two. The position in the 3D plot gives the relative rotation between the two axis distributions around the three rotational degrees of freedom (α, β, and γ). The entire range of all possible rotations was tested with a spacing of 15°, but only the results lying along the orthogonal rotational degrees of freedom are shown for clarity.

**Figure 7:**
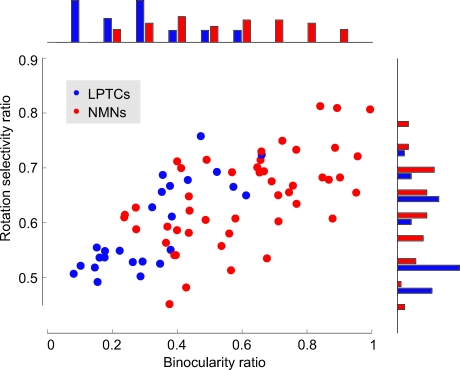
NMNs Are More Binocular and More Selective for Rotation Than LPTCs The rotation selectivity ratio is plotted against the binocularity ratio for each NMN (red) and LPTC (blue). A binocularity ratio of 1 means that the neuron was equally sensitive to motion in both visual hemispheres, a value of 0 means that the neuron only responded to motion in one hemisphere. A rotation selectivity ratio of 0 means that we estimate the neuron only responds to translation-induced optic flow, 0.5 means we estimate the neuron responds to translation and rotation equally, 1 means the neuron only responds to rotation. See Materials and Methods for details on how the ratios were calculated. Histograms at the top and side show the distribution of binocularity and rotation selectivity ratios across the NMN and LPTC populations. The bin size is 0.1 for the binocularity histogram and 0.04 for the rotation selectivity histogram. For information on which data point corresponds to which cell type, see Figure S2.

Here we show example NMN receptive field maps that illustrate the range of different map types obtained from each nerve (Figures 3–5) - population data are shown later (Figures 6,7,S1 and S2). The receptive field maps consist of local vectors plotted against azimuth and elevation. The azimuth and elevation indicate the horizontal and vertical angular position of a motion stimulus within the fly's visual field. The fly was oriented so that it was facing zero degrees azimuth and elevation. The orientation and length of each local vector in the receptive field maps indicate the NMN's local preferred direction of visual motion and relative motion sensitivity, respectively.

All receptive field maps, except where noted, are plotted with respect to recordings from the left part of the nervous system. Maps derived from NMN recordings in the right part of the nervous system are mirror-transformed. To distinguish between the different examples we present, we have labeled them, e.g., FN NMN A, FN NMN B, etc. This naming convention is for convenience only and should not be taken to imply that the data come from any particular identified neuron.

### Frontal Nerve NMNs

The FN is the biggest of the four neck motor nerves. NMNs running through this nerve innervate a variety of different neck muscles that, based on their anatomy [25], could potentially be involved in head inclination (nose-up pitch), declination (nose-down pitch), unilateral adduction (yaw), and unilateral depression (roll). FN-innervated muscles may potentially also be involved in head extension/retraction—the only possible translational degree of freedom of the head [25]. Milde et al. [23] found that FN neurons were tuned to a variety of directions in the frontal visual field and to downward visual motion laterally—from this they concluded that FN units were sensitive to roll. Using our receptive field mapping approach, we were able to determine which self-motions the FN units were most sensitive to with a higher degree of accuracy. We found FN units with receptive fields that appear to be tuned for pitch, roll and pitch-roll intermediates.

Our first FN NMN example, FN NMN A (Figure 3A), had a bilaterally symmetric receptive field with strong motion sensitivity in areas above the eye equator. In the frontal visual field, this NMN was most sensitive to vertical upward motion. Its directional preferences gradually became horizontal within the lateral visual field and eventually turned into a preference for almost vertical downward motion in the caudolateral visual field. The directional preferences were oriented along concentric circles around an area of low sensitivity at roughly ±90° azimuth. Such sensitivity minima, or singularities, within the receptive fields indicate the orientation of the neuron's “preferred rotation axis”, i.e. the axis about which the animal has to turn in order to most strongly activate the neuron. The rotation axis that this NMN preferred nearly coincides with the animal's transverse body axis, suggesting that FN NMN A would respond most strongly when the fly performs a nose-down pitch rotation.

FN NMN B (Figure 3B) responded most strongly to oblique upward motion in the frontal visual field above the eye equator and was slightly less sensitive to motion in the right visual hemisphere. The global appearance of this NMN receptive field was similar to that of the VS8 LPTC [17]: its preferred directions being oriented around a singularity at an azimuth of roughly −70°. Thus, the preferred rotation axis of FN NMN B lay between the longitudinal and transverse body axes, indicating that this NMN would probably respond best to nose-down pitch in combination with a slightly smaller roll component. Simultaneous recordings were often made from multiple FN NMNs with receptive fields similar to FN NMN B (Figure 3B).

FN NMN C (Figure 3C) had a binocular receptive field that covered the entire area tested in our experiments. The receptive field resembled an optic flow field generated during roll-rotation in combination with a subtle nose-down pitch. Accordingly, the NMN's preferred rotation axis was close to the fly's longitudinal body axis but slightly tipped downward frontally.

### Ventral Cervical Nerve NMNs

The VCN hosts three NMNs innervating different oblique horizontal (OH) muscles. These muscles were suggested to be involved in the control of yaw rotations of the head [25].

VCN NMNs (Figure 4A) were recorded from the OH muscle group. They responded to motion across an area that covered the ipsilateral visual hemisphere. In contrast to most other NMNs, the VCN NMNs did not appear to receive contralateral input beyond the region of the eye's binocular overlap [26].

Yaw rotation of the fly results in horizontal motion in the same direction across both eyes, whereas forward translation motion of the fly results in horizontal motion in opposite directions either side of the focus of image expansion. Therefore, to distinguish between yaw rotation and translation, a neuron needs a binocular receptive field. In contrast to most of the other NMN receptive fields, the VCN NMN receptive field was mostly monocular. This type of NMN may therefore respond to both yaw rotation and translation of the fly. Besides potentially controlling yaw head movements, such NMNs may also be involved in the control of head retraction. In principle the oblique horizontal muscles which VCN NMNs innervate [25] are suited to serve this function when simultaneously activated on either side of the neck motor system.

### Anterior Dorsal Nerve NMNs

The ADN contains only two motor neurons, both of which supply the transversal horizontal (TH) muscles involved in rotations of the head around the vertical axis, i.e., yaw [25].

ADN NMN A (Figure 4B) was recorded from the TH muscle group. It had the smallest receptive field of all the NMNs presented in this account. The extent of the receptive field nearly reached 90° along the azimuth, and the neurons' sensitivity dropped sharply toward the dorsal and ventral visual field.

Other ADN receptive fields, such as ADN NMN B (Figure 5A, also recorded from the TH muscle group), covered the entire area of the visual field examined. In a manner reminiscent of the HSE LPTC receptive field (Figure 5B), such ADN NMNs showed the highest sensitivity to horizontal motion along the entire eye equator (Figure 5A). Their motion sensitivity decreased toward both the dorsal and the ventral parts of the visual field. The singularity within this receptive field, though difficult to see in the cylindrical projection, lay at about +45° azimuth and +75° elevation, suggesting that this type of NMN would respond strongly during yaw rotation of the animal to the right.

### Cervical Nerve NMNs

The CN contains several neck motor neurons that, among others, innervate muscles involved in head declination [25]. Some of the units recorded from the CN were only sensitive to vertical downward motion in a very small portion of the frontal visual field (unpublished data). Because of their small receptive fields, the tuning of these units to self-motion could not be determined. We therefore excluded these units from the current analysis.

Wide-field CN NMNs were particularly sensitive to vertical downward motion in the frontal-to-frontolateral aspect of the contralateral visual field with a singularity at an azimuth of about −45° (Figure 5C). The finding that the CN NMNs respond most strongly in the contralateral field is in agreement with Milde et al. [23]. The CN NMNs were noticeably similar to the contralateral VS3 LPTC (Figure 5D) [17,27]. The distributions of the preferred directions and motion sensitivities within the CN NMN resembled optic-flow fields generated during nose-up banked turn to the left.

### Comparing Preferred Rotation Axes of NMN and LPTC Populations

The preferred rotation axis of a neuron is a convenient parameter to compare how the LPTC and NMN populations encode and control self-rotations. The preferred rotation axis is the body axis about which the fly would have to rotate to maximally excite a given neuron [28]. We quantitatively estimated the preferred rotation axes of all 47 NMNs studied and a group of 28 LPTCs for which the binocular receptive field maps had previously been obtained [27,29]. This was done by finding for each LPTC and NMN the rotation axis the animal would have to turn around to generate an optic flow field best matching the cell's receptive field map (see the Materials and Methods section). A second set of axes was obtained in the same manner from the mirror transformed NMN and LPTC receptive fields to account for the LPTCs and NMNs on the other side of the nervous system. This duplication of the dataset was based upon the well-supported assumption that bilateral symmetry exists in the LPTC and NMN populations [16,25].

Figure 6 shows the preferred rotation axes of the LPTCs and NMNs in blue and red, respectively. Each arrow in Figure 6B corresponds to the axis about which a fly would have to rotate clockwise to maximally stimulate a given neuron. For comparison, Figure 6A shows a fly oriented in the same coordinate system. To show the preferred rotation axes on all sides of the sphere, we also plotted the axes against azimuth and elevation in a 2D cylindrical projection (Figure 6C). The axes in the dorsal and ventral pole region of the cylindrical projection appear to be more scattered than they are in the spherical presentation (Figure 6B). This distortion is due to the reduction of dimensions when transforming the data from the 3D spherical representation to a 2D cylindrical projection.

To quantify the relationship between the LPTC and NMN preferred rotation axes, we binned the two axis distributions into equi-angular bins (15° in azimuth and elevation at the equator) and then performed a normalized spherical cross-correlation [30] between the two (Figure 6D). The color values in Figure 6D indicate the correlation coefficient, *r*, between the binned NMN and LPTC preferred axes at different relative rotations between the two sets of axes. All possible combinations of the three rotational degrees of freedom (α, β, and γ) were tested with a resolution of 15°. There was a clear peak in the cross-correlation function at zero rotation (Figure 6D), indicating that there was no systematic rotation between the NMN and LPTC preferred axes. At zero rotation, the correlation coefficient between the binned NMN and LPTC axes was *r* = 0.57. Thus, almost a third (coefficient of determination *r* ^2^ = 32%) of the variation in the measured NMN preferred rotation axes was related to the preferred axes of the LPTC population.

### Binocularity and Rotation Selectivity

The most obvious difference between the receptive fields of many NMNs and LPTCs is that, while the VS LPTCs tended to have monocular receptive fields [17,27], most NMNs responded to motion in both visual hemispheres. We quantified the degree of binocularity for each receptive field (Figure 7) by taking the ratio between the mean motion sensitivity in the left and right hemispheres (see the Materials and Methods section). The resulting binocularity ratio was 0.6 on average for the NMNs compared to 0.32 for the LPTCs. This increase in binocularity reflects a statistically significant difference between the two populations (*p* < 10^−8^, Student's two-sample *t*-test).

Theoretical analysis has shown that sampling both visual hemispheres enables a system to distinguish more accurately between the rotation- and translation-induced components of optic flow [31]. We investigated whether the increased binocularity of the NMNs was also accompanied by a higher rotation-selectivity than found for the more monocular LPTCs. To do so, we quantitatively estimated for each neuron its rotation selectivity ratio, which indicates the preference for rotation over translation based on the neuron's receptive field organization (see Materials and Methods). The rotation selectivity ratio ranges from 0 to 1, where a ratio of 0 indicates the neuron was selective for only translation, 1 indicates selectivity for pure rotation, and 0.5 results from an equal selectivity to rotation and translation. For both the NMNs and the LPTCs, the more-binocular receptive fields tended to have a higher selectivity for rotation (Figure 7). On average, the NMNs were more rotation-selective than the LPTCs. The LPTCs had a median rotation selectivity of 0.55 compared to a value of 0.65 for the NMNs. We found this 18% increase in rotation selectivity to be statistically significant (*p* < 0.01, Mann-Whitney U test). When the NMN receptive fields were artificially made to be monocular by setting the motion sensitivity on their weakest side to zero, the distribution of rotation selectivities was no longer statistically different from those of the LPTCs (*p* > 0.7, Mann-Whitney U test, median “monocularized” NMN rotation selectivity = 0.58). This suggests that the increased binocularity seen in the NMNs leads them to be more selective for rotation than the LPTCs.

## Discussion

In this study, we characterized the visual receptive field maps of NMNs in the fly and compared the results to those of their input elements, the LPTCs. As with the LPTCs, a general feature of the NMNs is the similarity of their receptive field maps to optic flow fields arising from self-rotations of the fly (Figures 3–5). In several cases, the NMN and LPTC receptive fields appear tuned to similar self-rotations (Figures 5 and 6). We show that the NMNs have a greater degree of binocularity than the LPTCs and quantitatively estimate that this makes them more selective for optic flow induced by rotation as opposed to translation (Figure 7). We propose that this increased binocularity reflects a simple “read-out” mechanism employed by the NMNs, in which they combine LPTC inputs from both sides of the brain to selectively extract the rotation information needed for gaze stabilization.

### Differences in Binocularity between NMNs and LPTCs

The NMN and LPTC receptive fields differ in how sensitive to motion they are at different positions in the visual field. In particular, most NMNs display a strong degree of binocularity, whereas their input elements, the LPTCs are either mostly monocular or weakly binocular [17,27,29]. This is especially true of those NMNs that receive LPTC inputs indirectly via descending neurons (FN and ADN NMNs [21]), possibly reflecting a high degree of binocular integration at the level of descending neurons. Such binocular integration has recently been found in at least one descending neuron that provides input to NMNs [32,33]. In particular, the visual response properties of the descending neuron DNVOS2 [33] are similar to some of the FN receptive fields we obtained (Figure 3C).

Binocular information is required to distinguish between the optic flow resulting from certain rotations and translations. Thus, by appropriately combining LPTC inputs to generate binocular receptive fields, NMNs can “read out” rotation information from the LPTC population while reducing the influence of translation-induced signals (Figure 7). This comparatively simple mechanism may be facilitated by some LPTCs already having receptive fields and therefore preferred axes of rotation similar to those the NMNs require to control head rotations (Figures 5 and 6). Such a straightforward mechanism would explain why the circuitry connecting LPTCs to NMNs is comparatively simple and direct [25], contributing to fast gaze stabilization responses [22].

### Potential Limitations

Our data provide an extensive survey of the NMNs that are sensitive to wide-field motion. However, it is possible that some visually responsive NMNs may have been missed in this study. For example, no NMN receptive fields similar to those of the VS5–6 LPTCs were found. The reason for this could be that no such NMNs exist, that we did not record from them due to a sampling bias, or that the visual responses of such neurons are gated by another sensory modality [34]. It is also possible that we may have recorded from NMNs with receptive fields similar to the LPTCs more often, while missing NMNs with very different receptive fields. If true, this would have artificially strengthened the correlation we find between the preferred axes of the LPTC and NMN populations (Figure 6D). We have, however, sampled units from all neck nerves (Figures 3–5). Furthermore, in some nerves we found multiple types of receptive fields, strongly suggesting that we have recorded from multiple types of neurons. In some cases, we have obtained simultaneous recordings from multiple units with similar receptive fields, indicating that we have been sampling separate units even among those with similar response properties. Thus, we are confident that the data are a thorough representation of the subpopulation of NMNs that are sensitive to wide-field motion.

In Figure 6, we describe the NMN and LPTC populations in terms of their preferred axes of rotation (see the Materials and Methods section). This is not a complete description of the cells' responses to self-motion, because the LPTCs [19,20], and possibly some NMNs (Figures 4A and 7), can also respond to translation-induced optic flow. However, as the neck motor system mainly compensates for rotations, it is reasonable for the purposes of this study to focus on the rotational component of the NMN and LPTC receptive fields. Furthermore, the preferred axis of rotation does not appear to be affected by translation. Karmeier et al. [28,35] have shown that the preferred rotation axis estimated from VS LPTC receptive fields is in agreement with that obtained through wide-field stimulation of the same neurons, even when rotation and translation are superimposed.

In describing the coordinate system made up of the NMN preferred rotation axes (Figure 6), we now have a more complete picture of the visuomotor transformation that takes place in the gaze-stabilization system of the fly. However, the NMN coordinate system we describe is based on the visual receptive fields of the NMNs, not the pulling planes of the muscles they innervate. And it is the pulling planes of the muscles that represent the final stage of the visuomotor transformation. Gilbert et al. [24], on the other hand, monitored head movements resulting from whole-nerve stimulation. Their results suggest a direct relationship between NMN preferred axes of rotation and muscle pulling planes, though this is not necessarily the case in other systems [5,36].

### Comparison to Previous Studies

In a previous pioneering study, Milde et al. [23] investigated the visual response properties of NMNs in the context of the anatomical organization of the neck motor system [25]. While our results on the local motion preferences of the neck motor neurons are generally compatible with those of Milde et al. [23], there are two decisive differences: (i) Milde et al. did not obtain the complex receptive field organization of the NMNs, limiting themselves to a description of each neuron as tuned to horizontal or vertical motion, and (ii) for many of the NMNs, they did not report the contralateral input that we find.

Here we provide a quantitative description of how the receptive fields of NMNs are adapted to specifically control certain head rotations, which significantly adds to the previous level of understanding [23]. The detailed knowledge about the receptive field organization of the NMNs, including their estimated preferred rotation axes, enables us to make specific predictions about the functional organization of the neck motor system which can be tested in future experiments. Based on the NMNs' and LPTCs' receptive field organization, future theoretical studies may be able to predict the connections between these two neural populations. Furthermore, our estimate of the motor neurons' preferred rotation axes should provide some additional guidance when investigating the actual pulling planes of the neck muscles.

### How Does the Visuomotor Transformation Take Place?

Directional motion information is acquired locally in retinal coordinates defined by the orientation of the hexagonal rows of the ommatidial eye lattice [8,37]. At this stage, the information is ambiguous with respect to self-motion, because different self-motions can result in the same direction of local motion [17]. VS LPTCs resolve this ambiguity by integrating local motion information from across one entire eye so that each neuron is broadly tuned to a specific axis of rotation [4,17,18]. Many of the resulting LPTC receptive fields are already similar to those required by the NMNs. Thus, the visual system LPTCs play a key role in the visuomotor transformation in that they convert local sensory information into signals related to self-motion, in this case rotation, which can immediately be used by various motor systems. However, the outputs of LPTCs are still partially ambiguous with respect to self-motion. LPTCs mostly respond to monocular inputs, but binocular motion information is needed to unambiguously distinguish certain rotation and translation components [31,38]. The NMNs perform this final step by integrating LPTC inputs from either side of the brain. We conclude that the fly gaze-stabilization system is an excellent example of task-specific processing of visual motion information that results in a simplified sensorimotor transformation.

## Materials and Methods

### Dissection and electrophysiology.

We mounted female 1–3-d-old blowflies (*C. vicina)* from the Department of Zoology, University of Cambridge, either dorsal or ventral side up on custom-made holders. The wing bases were waxed and the legs and wings removed. The resulting wounds were sealed with beeswax to reduce fluid loss. We aligned the orientation of the fly's eye with the center of the visual stimulus apparatus according to the deep pseudopupil [39], and then fixed its head in position with beeswax. The ocelli were obscured with nontoxic acrylic black paint.

In those experiments where the fly was mounted ventral side up, we cut a small window in the neck or thorax cuticle exposing the neck nerve to be studied. Two hook electrodes constructed from 0.025-mm-diameter silver wire were placed under the neck nerve of interest. Haemolymph was temporarily removed from the recording site and replaced with a mixture of petroleum jelly and paraffin oil. The tissue was kept moist with fly saline [15]. In those experiments where the fly was mounted dorsal side up, we used the same methods except that we placed the hook electrodes under neck muscles instead of a nerve, so we could record from NMN axon terminal arborizations. Figure 2C shows an example of the characteristic signal structure we obtained in such recordings: a fast NMN action potential was reliably followed in a 1:1 fashion by a slower biphasic potential, most likely the induced neck muscle potential. In all, we recorded from 47 NMNs in 38 flies that responded to visual motion over an area wider than 90° along the azimuth.

Signals from the hook electrodes were amplified 3000× by a Brownlee Precision amplifier Model 440 operating in differential AC mode. A PC-controlled National Instruments PCI-6025E data acquisition board sampled the amplifier output at 10 kHz. Software for stimulus control, data acquisition, spike sorting by template matching, and data analysis was programmed in Matlab (Mathworks).

### Visual stimuli for mapping receptive fields.

Visual stimuli were presented on a green cathode ray tube (CRT, P31 phosphor) driven by an Innisfree Picasso image synthesizer at a refresh rate of 182 Hz. We placed the CRT 7.4 cm or 18.5 cm from the fly so that the circular screen aperture subtended a visual angle of 62.6° or 27.3°. Depending on the visual responsiveness of the unit being studied, one of two different types of visual stimuli was used to obtain directional tuning curves at 66 different positions within the NMNs' receptive fields. For NMNs that were highly sensitive to local visual motion, we used a procedure introduced by [40]. In brief, a black dot (7.6° diameter) was moved on a circular path (10.4° diameter) across a green background (96% contrast) completing 6 cycles at a speed of 2 cycles/s. By traveling on a circular path, this stimulus covered all possible directions of visual motion. Correlating a unit's change in spike rate with the direction of dot movement revealed the local directional tuning curve. See [40] for further details.

If the recorded unit did not produce a robust response to the dot stimulus, we used square wave visual gratings instead. The gratings had a 96% contrast, spatial wavelength of 10° and were moved perpendicular to their orientation with a temporal frequency of 5 Hz across the full extent of the 62.6° diameter screen. The grating was moved in 16 different directions with a spacing of 22.5°, presented in a pseudo-random order. Before each motion stimulus, a blank screen of the same mean luminance as the grating (18 cd/m^2^) was shown for 5 s and the neuron's baseline spike-rate recorded. In these experiments, we defined the response to a grating as the mean spike rate during a 1-s stimulus presentation minus the baseline spike rate. As an example, Figure 2A and 2B show the response of a CN NMN to grating motion in opposite directions. Plotting the responses to visual motion against the 16 different directions of motion revealed the neuron's local directional tuning curve. From the tuning curve, we could estimate the local preferred direction and motion sensitivity by finding the phase and amplitude of the fundamental harmonic in a fast Fourier transformation of the tuning curve (Figure 2D).

The CRT was mounted upon a semicircular frame that allowed it to be moved to different positions within the fly's visual field. The CRT position could range from −120° to +120° in azimuth and −70° to +75° in elevation. We obtained directional tuning curves in a pseudo-random order at different positions within the fly's visual field. For elevations 15° and −15° relative to the horizontal plane of the stimulus apparatus, we presented visual stimuli with 15° spacing along the azimuth. For elevations of 45/ −45° and 75/ −70°, we used an azimuthal spacing of 30° and 45°, respectively.

Once we had determined the local preferred direction and local motion sensitivity for a unit at multiple locations within the visual field, we plotted them in vector field maps of the visual field where each position is defined by its azimuth and elevation. The orientation and length of each vector indicate the neuron's local preferred direction and motion sensitivity at each point in the visual field. Figure 2E and 2F show the receptive field map for an individual CN neck motor neuron as obtained with the dot method and the grating method, respectively. The boxed arrow in Figure 2F is derived from the tuning curve in Figure 2D.

While the global distribution of directional motion preferences looks similar in the maps obtained with the dot method and the grating method, there are two differences at the local scale: first, in the grating map, the position-dependent changes of local preferred directions appears to be more smooth, and second, the area of higher local motion sensitivity seems to be extended (Figure 2E and 2F). Both of these differences are to be expected, since the grating stimulus covers a larger visual angle and therefore the upstream cells will be integrating signals over more directional selective input elements, which results in a general smoothing of the receptive field map. Despite these differences in detail, both methods produced similar results with respect to the neuron's estimated preferred rotation axis (see below for details on the estimation procedure).

In all receptive field maps presented, black arrows show experimentally determined results whereas gray arrows result from interpolation. The spline interpolation method used is described by [41]; it makes no assumptions other than that the transitions between data points are smooth.

The lowest position at which the CRT could be held was −70° in elevation, whereas the highest position was 75°. Thus, a vertical asymmetry exists between maps taken from flies mounted dorsal and ventral side up. To allow the comparison of receptive fields obtained in different experiments, this asymmetry was overcome by performing a 5° extrapolation [41] on the data taken at an elevation of −70°. Consequently, instead of plotting the data obtained at −70° elevation, we plot extrapolated data at −75° elevation. Receptive field maps taken from flies mounted ventral side up were flipped vertically to allow comparison with maps taken from flies mounted dorsal side up.

### Measuring binocularity and rotation selectivity.

The receptive fields we describe range from monocular, i.e., only responding to motion in one visual hemisphere, to fully binocular, i.e., responding to visual motion across both hemispheres. To describe the degree of binocularity of each neuron, we computed a “binocularity ratio” for each neuron's receptive field. For each neuron, we computed the mean motion sensitivity in the left and right visual hemispheres. The binocularity ratio is simply the smaller of the two mean motion sensitivities divided by the larger. A value of 1 means that the neuron was equally sensitive to motion in both visual hemispheres, a value of 0 means that the neuron only responded to motion in one hemisphere. All the binocularity ratio data passed the Lilliefors test for normality, so parametric statistics are used to describe these data. The NMN and LPTC binocularity ratio data sets did not pass an F-test for equal variances, so we used Student's 2-sample *t*-test with a correction for unequal variances to compare the two data sets.

To quantitatively estimate each neuron's selectivity for rotational versus translational motion, we applied the iterative least-square algorithm developed by Koenderink and van Doorn ( KvD [38]) to each neuron's receptive field map. This procedure parameterizes each neuron's receptive field by its preferred self-rotation and translation components vectors **R** and **T**; similar approaches have been used previously for the LPTCs [29]. The magnitudes of the two components of the parameterization, |**R**| and |**T**|, give the relative contribution of the rotation and translation parameters when calculating an optic flow field that best matches a neuron's receptive field. Thus, |**R**| and |**T**| can be thought of as representing how much of the receptive field can be explained by rotation and translation respectively. We used the magnitude of the KvD rotation and translation components to calculate the proportion of the receptive field that was explained by the rotation component:

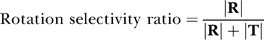



A rotation selectivity ratio of 0 means that we estimate the neuron only responds to translation-induced optic flow, 0.5 means we estimate the neuron responds to translation and rotation equally, 1 means the neuron only responds to rotation. The rotation selectivity ratio data sets did not pass the Lilliefors test for normality, so we use nonparametric statistics to describe these data. Binocular receptive field data were used for all calculations. For all metrics, we only used data from receptive field positions where data were available for both the LPTCs and NMNs.

### Estimating preferred axes of rotation.

The receptive field maps allow us to compare the visual responses of individual cells. To compare the entire coordinate system used by the VS and HS cells for encoding self-rotation to the coordinate system used by NMNs for controlling head rotation, we estimated each cell's preferred axis of rotation. This was done by comparing a cell's receptive field to optic flow fields generated by rotations about different axes. The rotational flow fields were computed using the formalism described by Koenderink and van Doorn [38]. The local motion vectors making up a given optic flow field (***P***) were projected onto the local preferred directions of the cell's receptive field (***U***). The resulting values were weighted by the cosine of their elevation θ_*j*_ to compensate for the over-sampling of high and low elevations and then summed across all locations *j*, where *N* is the total number of local preferred direction measurements. The resulting value *s* is a measure of the similarity between the cell's receptive field and the rotational optic flow field [29]:

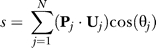



We repeated this procedure for axes of rotational optic flow across the entire sphere with a spacing of 1° between axes tested. We defined the cell's “preferred axis of rotation” as the axis of self-rotation that resulted in optic flow most similar to the cell's receptive field, and therefore the largest value of *s*. This definition is based on the assumption that the more similar an optic flow field is to a unit's receptive field map, the stronger the unit's response to the optic flow field will be [28,35]. Using this method, we obtained a preferred axis for all LPTCs and NMNs. The preferred axis is defined by just two angles, its azimuth and elevation, allowing easy comparison of a large number of cells simultaneously.

## Supporting Information

Figure S1 Preferred Rotation Axes of NMNs and LPTCs The data from Figure 6C are replotted to allow identification of the cell type from which each preferred rotation axis was derived. Solid symbols indicate LPTCs, the color and shape of the symbol represents the type of cell from which the plotted axis was derived. Open symbols indicate NMNs, the color and shape of the symbol represents the nerve from which the NMN recording was made.
(2.67 MB EPS)

Figure S2 Binocularity Versus Rotation Selectivity The data from Figure 7 are replotted to allow for an identification of the cell type from which each data point was derived. LPTCs are plotted in solid symbols, the color and shape indicates the VS or HS cell type. NMNs are plotted in open symbols, the symbol color and shape indicating from which nerve the NMN recording was obtained.
(2.89 MB EPS)

## Abbreviations

ADN: anterior dorsal nerve
CN: cervical nerve
CRT: cathode ray tube
FN: frontal nerve
HS: horizontal system
LPTC: lobula plate tangential cell
NMN: neck motor neuron
OH: oblique horizontal
TH: transverse horizontal
VCN: ventral cervical nerve
VS: vertical system
